# Correlation between clinical indices and findings of joint ultrasonography in juvenile idiopathic arthritis with adalimumab therapy

**DOI:** 10.1186/1546-0096-9-S1-P206

**Published:** 2011-09-14

**Authors:** Ryoki Hara, Tomoyuki Imagawa, Takuma Hara, Masako Kikuchi, Takako Miyamae, Masaaki Mori, Shumpei Yokota

**Affiliations:** 1 Yokohama City University School of Medicine Department of Pediatrics, Kanagawa, Japan

## Background

Usefulness of adalimumab (ADA) therapy in patient with rheumatoid arthritis has already established. But the efficacy of ADA in patient with juvenile idiopathic arthritis (JIA) and joint ultrasonography (US) findings of them are still unknown.

## Aim

To evaluate correlation of joint US findings and other clinical indices in JIA patient newly introduced ADA therapy.

## Methods

4 polyarticular JIA (p-JIA) children diagnosed as JIA according to ILAR criteria were enrolled. All were female. The median age was 12 years (range; 10 to 17 years), and median disease duration was 5.6 years (range; 2.5 to 7.5 years). Two patients were administered prednisolone and methotolexate, One prednisolone only, and one drug free, as preceeding therapy. ADA was administered biweekly at a dose of 40mg per body.

Demographic data were collected. Joint US examination was demonstrated at 0 (prior to administration of ADA), 4, and 24 weeks.

## Results

All of 4 patient had successfully continued ADA therapy for 24 weeks. There were no severe adverse events in this term. At 24 weeks after introduction of ADA, ACR pedi 50 were achieved by 3 patients. The average of MMP-3 at 0 weeks is 441.8 (56.1 – 690.8) ,and fell to 120.4 (45.5 – 185) after 24 weeks. R value of DAS28 and US grade of power doppler (grade 0 to 3) is 0.73.

## Conclusion

Efficacy of ADA therapy in children with JIA is demonstrated, and joint US is a good tool to evaluate disease activity of JIA.

